# Compressive and Shear Behavior of Masonry Reinforced with Ultra-Rapid-Hardening Fiber-Reinforced Mortar (URH-FRM)

**DOI:** 10.3390/ma15248825

**Published:** 2022-12-10

**Authors:** Joo Ha Lee

**Affiliations:** Department of Civil and Environmental Engineering, The University of Suwon, Hwaseong-si 18323, Republic of Korea; leejooha@suwon.ac.kr; Tel.: +82-31-220-2159

**Keywords:** masonry, earthquake, URH-FRM, compressive, shear, ductility

## Abstract

Masonry structures are very vulnerable to lateral forces such as earthquakes. In particular, for existing masonry buildings that have not been designed for earthquake resistance, appropriate seismic resistance retrofit is required. In this study, ultra-rapid-hardening fiber-reinforced mortar (URH-FRM), which has a high ductility, with an ultimate tensile strain of about 0.07, and is an economical and easy-to-construct seismic reinforcing material, was developed. Compressive strength and initial shear strength tests were performed on masonry prisms reinforced with the URH-FRM. As an experimental variable, the reinforcement thickness of the URH-FRM was varied from 10 to 30 mm and the structural performance was compared with specimens reinforced with general mortar and specimens without reinforcement. As a result, the beneficial effect of URH-FRM on the in-plane initial shear strength of horizontal bed joints in masonry prisms was confirmed. In addition, the thicker the URH-FRM reinforcement, the clearer the improvement in ductility through strain hardening.

## 1. Introduction

The Korean Peninsula is known as a moderate and weak earthquake zone, but, as can be seen from the magnitude 5.8 and magnitude 5.4 earthquakes that occurred in Gyeongju in 2016 and Pohang in 2017, respectively, it is no longer guaranteed that the Korean Peninsula is safe from earthquakes. As the frequency of intra-plate earthquakes is increasing recently, analysis has shown that there is potential for large-scale earthquakes in Korea [1]. According to the earthquake damage prediction model reported by Choi et al. [2], if a magnitude 7.0 earthquake occurs in Seoul, the total loss is estimated to be USD 126.6 billion. Therefore, preemptive earthquake disaster prevention measures are essential to minimize disaster damage. In Korea, government-level earthquake-resistance measures such as revision of design standards, allocation of budget for seismic retrofit, and incentive system for seismic retrofit of private buildings are being implemented, but so far, the earthquake-resistance rate of buildings has been very low [3].

Among various types of buildings, this study focuses on masonry structures. Since masonry has structural characteristics that are weak against lateral forces, it is very vulnerable to earthquakes. There are a lot of masonry buildings that are not designed to withstand earthquakes, and worse yet, many of them are in a state of structural deterioration due to aging. Therefore, there have been many studies on seismic reinforcement for such masonry buildings. Among the new reinforcement methods, steel, steel stripes [4,5], steel-bar truss [6], dry-connected steel plate frames [7], etc., have been recently introduced. However, the steel reinforcement method still has some disadvantages such as high cost, difficulty in installation, and increased self-weight. More recently, fiber-reinforced polymers (FRPs) have been used instead of steel to reinforce masonry. These new composite materials are typically composed of fibers such as carbon (CFRP), glass (GFRP) or aramid (AFRP). Many experimental studies have shown that FRP reinforcement technology improves the seismic performance of masonry. However, FRP has several drawbacks such as high cost, low impact resistance, low fire resistance, application limitations in high and low temperature conditions, and debonding failure due to various causes [8,9,10,11].

Another technique for seismic reinforcement of masonry is to attach textiles made of long and continuous yarns to the masonry using cementitious materials such as mortar or concrete. There have been many studies on the improvement of the seismic performance of masonry using this composite material, called textile-reinforced mortar (TRM), textile-reinforced concrete (TRC), or fabric-reinforced cementitious matrix (FRCM), etc. [11,12,13,14,15,16,17,18]. However, the poor toughness of the matrix, serviceability, and utilization rate, which is the ratio of the actual strength of the textile to its theoretical value, still need to be improved [13]. The use of cementitious composites alone for seismic reinforcement of masonry began with the development of cementitious composites with high tensile strength and ductility, called engineered cementitious composites (ECC), ultra-high ductile concrete (UHDC), ultra-high-performance concrete (UHPC), etc. [19,20,21,22,23,24,25,26]. Soleimani-Dashtaki [27] developed an eco-friendly ductile cementitious composite (EDCC) with excellent ductility and toughness for retrofitting unreinforced brick masonry walls. The average tensile strength and strain capacity of the EDCC were 5.19 MPa and 3.24%, respectively. Chen et al. [24] performed shaking-table tests on 5-story masonry structures reinforced with ultra-high ductile cementitious composites (UHDCC), with the average tensile strength and strain of 3.9 MPa and 6.1%, respectively. In this study, ultra-rapid-hardening fiber-reinforced mortar (URH-FRM) with higher tensile strength and strain capacity (i.e., more than 6 MPa and 6%, respectively) than EDCC and UHDCC, which are new types of ECC, was used. The URH-FRM can be sprayed on the masonry wall, making it easy to construct. The compressive strength and shear performance of the masonry reinforced with URH-FRM were investigated to evaluate the possibility of its use as a seismic reinforcing technique.

## 2. Experimental Program

### 2.1. Materials

Clay bricks and joint mortar were used to manufacture the masonry specimen, and general mortar and URH-FRM were used as reinforcing materials. The mortar used for reinforcement was the same as the mortar for joints. The size of one clay brick is 190 mm, 57 mm and 90 mm in length, height, and depth. The compressive strength of the bricks measured according to KS L 4201 [28] is 30.6 ± 0.9 MPa (*n* = 5; 29.7, 30.4, 31.9, 29.9, 31.1 MPa). Both the brick joint and reinforcement mortars were prepared by mixing water with dry ready-mixed cement mortar on the market. The compressive strength of the mortar at 28 days of age measured according to KS L ISO679 [29] is 18.7 ± 1.0 MPa (*n* = 6; 17.4, 17.7, 18.8, 18.9, 19.2, 20.1 MPa).

**Figure 1:**
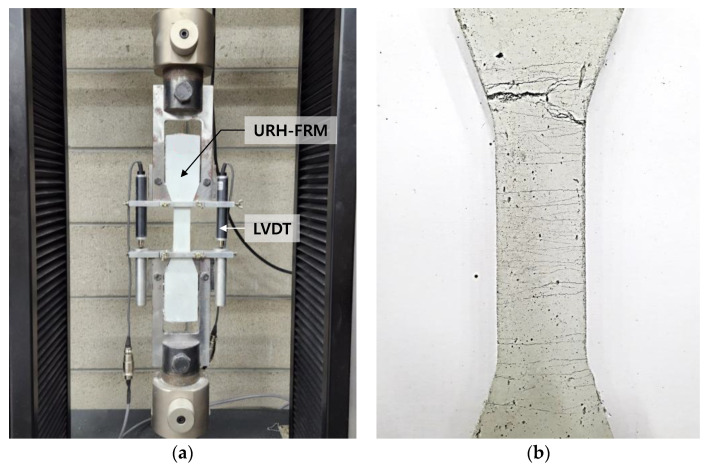
Direct tensile test of I-shaped URH-FRM: (**a**) Test setup; (**b**) URH-FRM specimen after testing.

**Figure 2:**
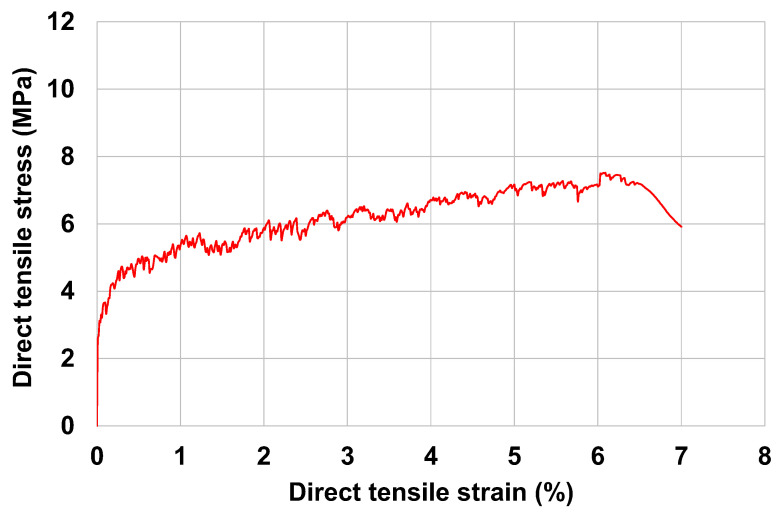
Direct tensile stress–strain curve of URH-FRM.

URH-FRM was first introduced by Chun et al. [30,31]. In this study, the URH-FRM mix proportions of Chun et al. [31] were modified. Table 1 shows the mix proportions of URH-FRM used in this study. Ultra-rapid-hardening (URH) cement was used instead of ordinary Portland cement as the base binder. In addition, ground granulated blast-furnace slag, limestone powder, cement kiln dust, and silica fume have been incorporated as supplementary cementitious materials. The chemical compositions of all cementitious material can be found in Chun et al. [31]. Coarse aggregate was not used, and silica sand with a diameter of 0.2 to 0.3 mm was used as fine aggregate. A polycarboxylate-based high-range water-reducing admixture (HRWRA) was used in the range of 1.5 to 2% of the total amount of binder to achieve a slump flow of 200 to 210 mm. Additionally, set retarder and viscosity modifying agent were incorporated. Polyethylene (PE) fibers were incorporated at 1.5% volume fraction and their properties are shown in Table 2. Figure 1a shows the direct tensile test setup of I-shaped (Dog-bone) URH-FRM specimens performed according to the JSCE recommendation [32]. As shown in Figure 1b, URH-FRM exhibited ductile behavior forming multiple microcracks under tension. Figure 2 shows the direct tensile stress–strain curve of URH-FRM. The maximum tensile strength and maximum tensile strain capacity of the URH-FRM were about 7.5 MPa and 7%, respectively. To the best of the knowledge of the author, this URH-FRM has the best tensile performance among the new types of ECCs.

### 2.2. Test Specimens

In this study, compressive strength and initial shear strength tests were performed on unreinforced, general mortar reinforced, and URH-FRM reinforced masonry specimens. In addition, the effect of the reinforcing thickness of URH-FRM on the structural behavior of the masonry was investigated. Table 3 shows the experimental parameters. The first and second letters and numbers in the specimens’ names indicate the type of test, reinforcing material, and the reinforcing thickness, respectively. For example, specimen CH10 represents a compressive strength test specimen reinforced with URH-FRM with a thickness of 10 mm. 

The thickness of the masonry joint is the same for all specimens: 10 mm. One side of the specimen was overlaid with a reinforcing material three days after the base masonry prism was made by stacking clay bricks. As a reinforcing material, URH-FRM was applied to the specimen surface by spraying, and general mortar was hand-applied with a trowel. When URH-FRM was sprayed with a thickness exceeding 30 mm on the wall of the specimen, it flowed down on its own. Therefore, the URH-FRM reinforcement thickness parameters were set to 10, 20, and 30 mm. The finished specimens including one-sided reinforcement were cured for 28 days under room conditions (temperature 24 ± 8 °C, relative humidity 25~75%).

### 2.3. Test Method

**Figure 3:**
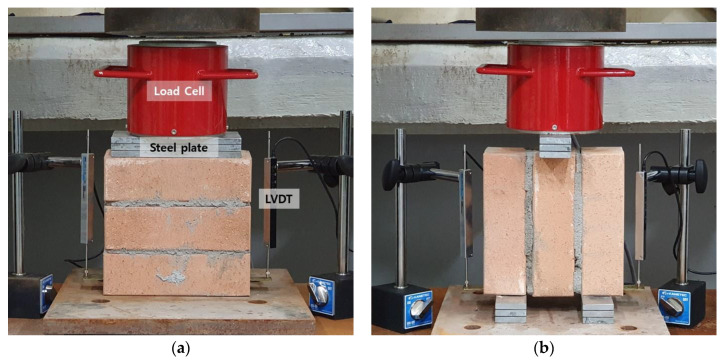
Test setup for compressive strength and initial shear strength: (**a**) Compressive strength test of specimen CR; (**b**) Initial shear strength test of specimen SR.

Figure 3 shows the experimental setup for compressive strength and initial shear strength of the masonry prism. The compressive strength test of the masonry prism was performed according to ASTM C1314-21 [33]. The in-plane initial shear strength of the horizontal bed joints in the masonry was measured according to BS EN 1052-3:2002 [34]. Three identical specimens were prepared for each experimental variable. The applied load and displacement were measured using a load cell and linear variable differential transformer (LVDT). Steel plates were used to help evenly distribute the applied load. The loading rate was 1.0 mm/min. The load and displacement measured by the load cell and LVDT were automatically recorded every second through the data logger.

## 3. Experimental Results and Discussions

### 3.1. Compressive Strength of Masonry Prism

According to ASTM C1314-21 [33], the compressive strength of the masonry prism was calculated by the following Equation (1):

(1) $$f_{p,c}=\frac{P}{A_{p,c}}\times \alpha$$

where, $f_{p,c}$: compressive strength of masonry prism (MPa), P: applied load (N), $A_{p,c}$: area of masonry prism (mm), α: compressive strength factor ($\alpha =h_{p}/t_{p}$), $h_{p}$: height of masonry prism (mm), and $t_{p}$: thickness of masonry prism (mm).

**Figure 4:**
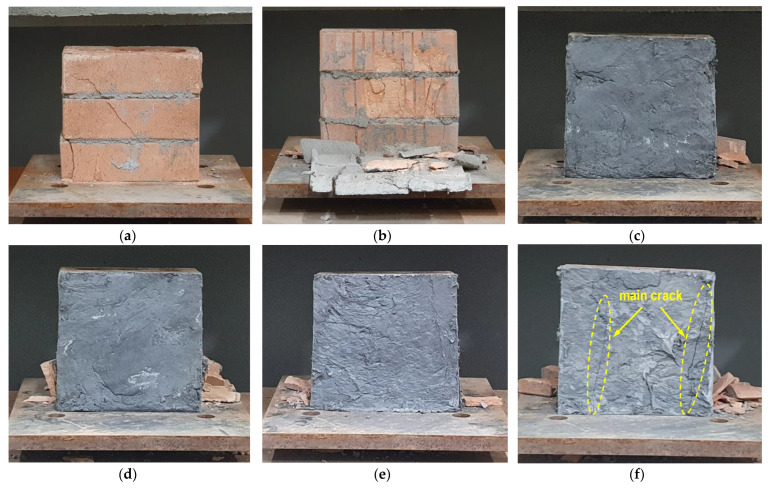
Failure mode for compressive strength test: (**a**) CR #1; (**b**) CM10 #1; (**c**) CH10 #1; (**d**) CH20 #1; (**e**) CH30 #1; (**f**) CH30 #3.

Table 4 summarizes the compressive strength test results for each variable composed of three specimens. The coefficient of variation of the CH30 test results was relatively large, which seems to be due to the difficulty in constructing the URH-FRM with 30 mm thick reinforcement, resulting in construction deviation between specimens of the same series. Figure 4 shows representative failure modes for each variable. As shown in Figure 4b, in specimen CM10, the overlaying general mortar cracked and fell off first, and then the bricks were broken. As a result, in terms of resistance to compression, it was no different than the unreinforced specimen CR. It is difficult to improve the compressive strength of the masonry prism with general mortar having a lower compressive strength than brick. As shown in Figure 4c–e, the CH series specimens showed no damage or only minor cracks in the URH-FRM and were fractured due to vertical or diagonal cracks in the bricks and joints of the unreinforced side. As shown in Figure 4f, unlike other CH series specimens, CH30 #3 had major cracks on the surface reinforced with URH-FRM and showed relatively greater compressive strength of masonry prism. This improvement in compressive strength is because the reinforced URH-FRM on one side suppressed the occurrence and propagation of cracks on the other unreinforced side and shared the load. However, according to the overall experimental results of the CH series specimens, the effects of URH-FRM and overlay thickness on the compressive strength were not consistent because the unreinforced side of the masonry prism governed the failure. Obviously, the compressive strength of the masonry prism will increase significantly if the URH-FRM is reinforced on both sides. However, URH-FRM is a specialized material for improving ductility through the excellent tensile performance of the material, and it is not desirable to use it for the purpose of improving compressive strength [30].

**Figure 5:**
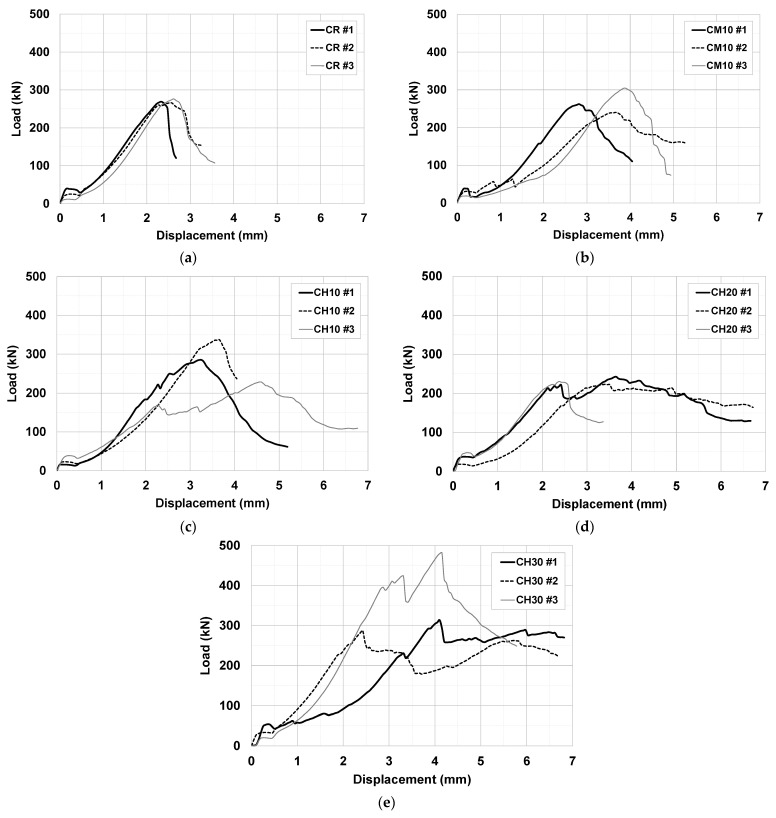
Load–displacement response of compressive strength test: (**a**) CR; (**b**) CM10; (**c**) CH10; (**d**) CH20; (**e**) CH30.

**Figure 6:**
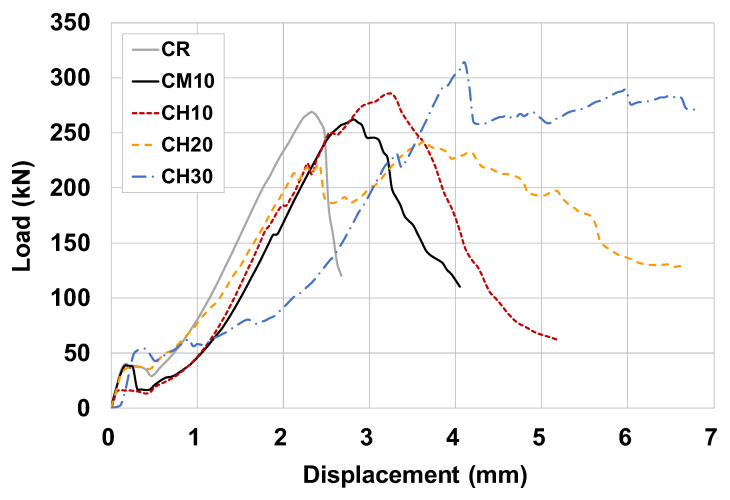
Comparison of load–displacement response of compressive strength test of specimen #1 for each variable.

Figure 5 shows the load–displacement response of the compressive strength test for all three specimens of each variable. As shown in Figure 6, the load–displacement response of specimen #1 for each variable was compared to investigate the difference in compressive behavior according to the reinforcing overlay material and thickness. The specimens reinforced with URH-FRM showed greater displacement at peak load than the CR and CM specimens. After the peak load, the CH specimens exhibited ductile behavior that fractured after significant displacement occurred. It is noteworthy that the thicker the reinforcing URH-FRM, the better the ductility with more pronounced strain hardening. Although the effect of URH-FRM and its reinforcement thickness on the compressive strength was insignificant, the beneficial effect on the post-peak ductile behavior was clear.

### 3.2. Initial Shear Strength of Masonry Prism

According to BS EN 1052-3:2002 [34], the shear strength was calculated by Equation (2):

(2) $$f_{p,s}=\frac{P}{2A_{p,s}}$$

where, $f_{p,s}$: initial shear strength of masonry prism (Mpa), P: applied load (N), and $A_{p,s}$: area of masonry prism (mm^2^).

**Figure 7:**
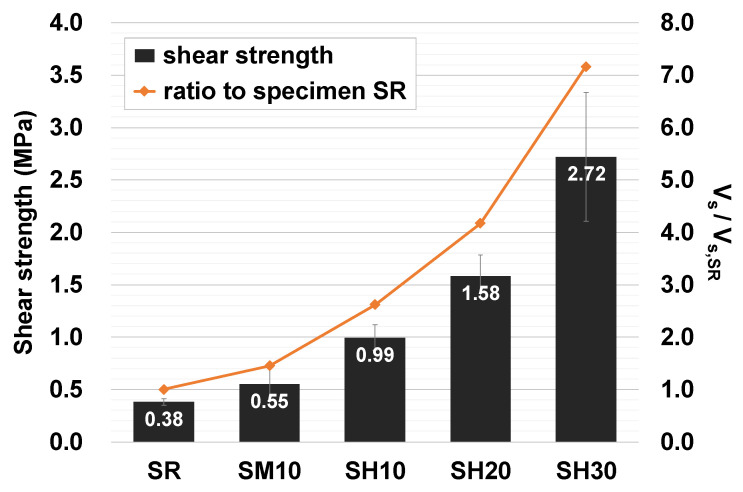
Results of initial shear strength of masonry prism.

Figure 7 shows the average shear strength of three specimens for each variable as a bar graph and the shear strength ratio compared to the SR specimen as a line graph. The beneficial effect of URH-FRM reinforcement on in-plane initial shear strength of horizontal bed joints in masonry prism was confirmed. The shear strength of the SH10 specimen was 2.6 times and 1.8 times greater than that of the SR and SM10 specimens, respectively. It was also observed that the shear strength increased as the reinforcement thickness of URH-FRM increased. The shear strengths of SH20 and SH30 were 4.2 and 7.2 times that of the reference specimen SR, respectively. 

**Figure 8:**
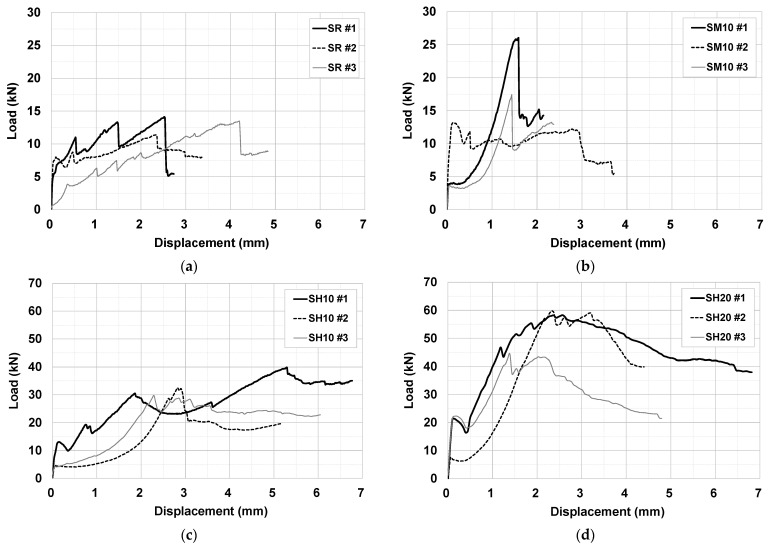

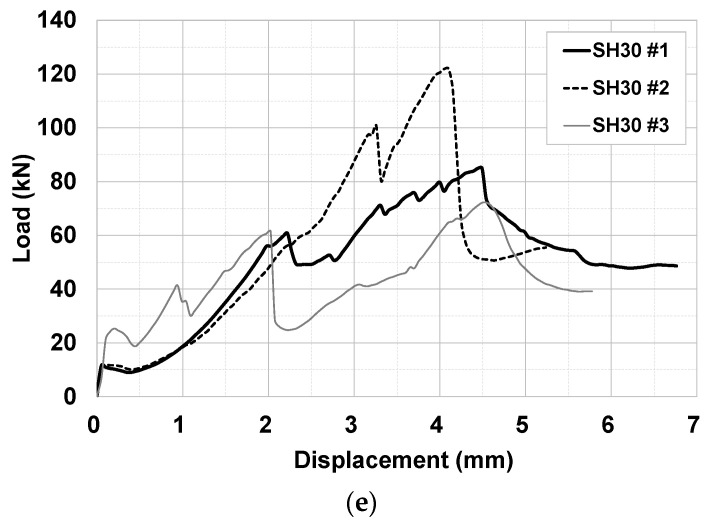
Load–displacement response of initial shear strength test: (**a**) SR; (**b**) SM10; (**c**) SH10; (**d**) SH20; (**e**) SH30.

**Figure 9:**
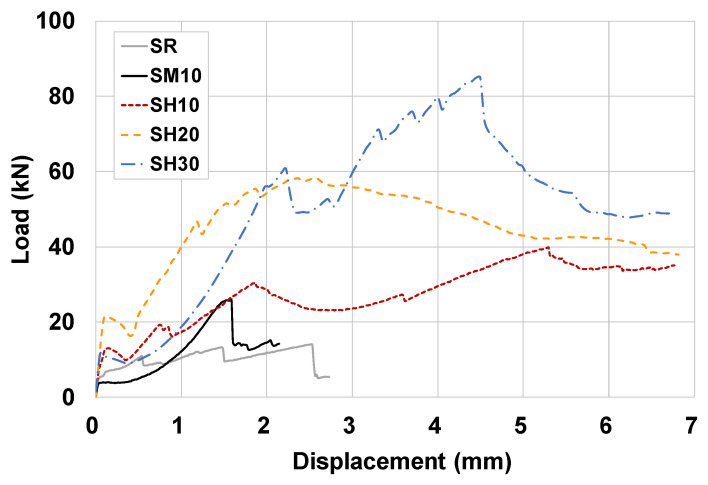
Comparison of load–displacement response of initial shear strength test of specimen #1 for each variable.

**Figure 10:**
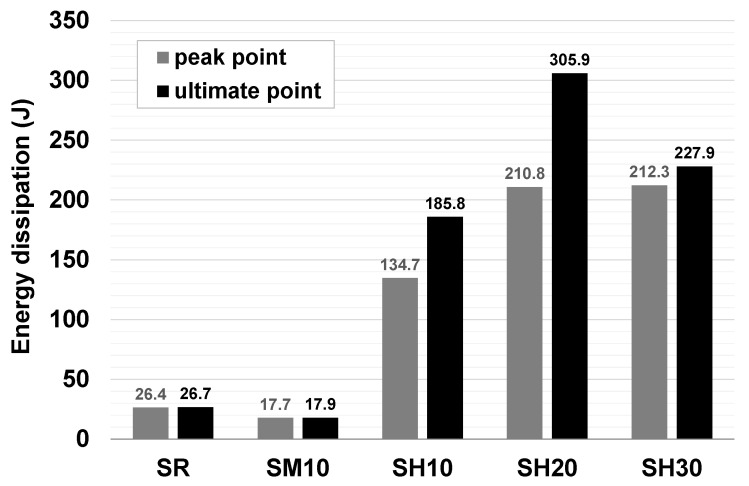
Energy dissipation capacity of specimen #1 for each variable.

Figure 8 shows the load–displacement response of the initial shear strength test for all three specimens of each variable. Figure 9 shows the load–displacement response of the initial shear strength test for a representative specimen #1 for each variable. The masonry prism reinforced with URH-FRM showed superior behavior not only in shear strength, but also in ductility. The SR and SM10 specimens showed abrupt brittle fracture after the main crack occurred, whereas the SH series specimens showed ductile behavior while continuing to support a certain amount of load and increasing the deformation even after the occurrence of major cracks or reaching the peak load. The greater the thickness of the URH-FRM reinforcement, the higher the shear strength, but, interestingly, the maximum deformations at failure were all at a similar level. Figure 10 compares the energy dissipation capacity at the peak load and ultimate load points. The energy dissipation capacity, which can be used to measure the ability of a specimen to deform without fracturing, was calculated as the area under the shear load–displacement curve. In the load–displacement response curve, the ultimate shear load point was defined as the point where 80% of the peak shear strength was reached after the peak load point [35,36,37]. Compared to the unreinforced specimen SR, the energy dissipation capacity of SM10 reinforced with general mortar was not improved, but the SH series reinforced with URH-FRM showed a clear improvement in energy dissipation capacity. SH20 showed about 8.0 times and 11.5 times greater energy dissipation than SR at the peak load and ultimate load points, respectively. It is interesting to note that SH20 and SH30 showed similar energy dissipation at the peak point, but at the ultimate point, SH20 showed 34.2% greater energy dissipation than SH30. This is partly due to better stress redistribution prior to peak loading in specimens reinforced with URH-FRM of appropriate thickness [36,38]. Similar to the results of this study, Soleimani-Dashtaki et al. [27] also reported that a single-sided EDCC retrofit of 20 mm thickness is sufficient for most low-rise school masonry buildings.

**Figure 11:**
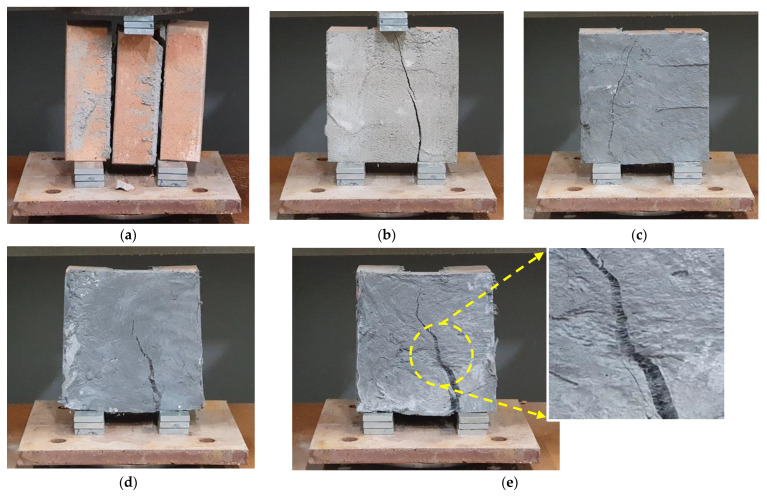
Failure mode for initial shear strength test: (**a**) SR; (**b**) SM10; (**c**) SH10; (**d**) SH20; (**e**) SH30.

The specimen failure mode in Figure 11 provides an intuitive understanding of why URH-FRM improves shear performance. In the unreinforced specimen SR, bond failure occurred at the interface between the brick and the joint mortar, which is a relatively weak part. However, if one side of the masonry prism is overlaid with a reinforcing material, the reinforcing material resists the interfacial fracture between the brick and the joint and bears the bending moment and shear force. However, in the case of SM10, the strength and ductility of the reinforcing mortar itself was poor, so the shear strength improvement of the masonry prism was insignificant and brittle failure could not be prevented. In the URH-FRM reinforcing specimens, a typical flexural-shear crack occurred, in which the crack initially generated by the bending moment on the inner surface of the support propagates in the diagonal direction by the combination of shear forces. As shown in Figure 11e, the fibers incorporated in URH-FRM bridged the cracks, which significantly contributed to the improvement of shear strength and ductility [31].

## 4. Conclusions

In this study, URH-FRM with world-class tensile performance (tensile strength 7.5 MPa, tensile strain 7%) was developed. One side of the masonry prism was reinforced by spraying the URH-FRM. Then, through the evaluation of the compressive and shear strength of the masonry prism, the applicability of URH-FRM as a seismic reinforcing material was investigated. In terms of compressive strength, the performance improvement could not be clearly confirmed, as the failure occurred on the unreinforced side of the masonry prism. If URH-FRM is used to reinforce both sides of the masonry prism, the compressive strength will increase, but at the same time the constructability and cost will be poor. It should also be noted that the main performance required as a seismic reinforcing material is not the enhancement of compressive strength. Unlike the compressive strength, the beneficial effect of URH-FRM reinforcement on in-plane initial shear strength of horizontal bed joints in the masonry prism was confirmed. The shear strength increased as the thickness of the URH-FRM reinforcement increased. URH-FRM not only improved the shear strength of the masonry prism, but also induced ductile behavior, as significant deformation occurred even after the appearance of major cracks or reaching the peak load. In terms of energy dissipation capacity, the optimum reinforcement thickness of URH-FRM is 20 mm. When reinforced with a thickness of 30 mm, the shear strength increased, but the energy dissipation capacity rather decreased, showing a backward ductile behavior. Moreover, URH-FRM thickness of 30 mm is relatively poor in terms of workability and economic feasibility. In order to utilize URH-FRM for seismic reinforcement of masonry, relevant studies including the structural behavior of full-scale masonry walls reinforced with URH-FRM should be sufficiently conducted.

## Figures and Tables

**Table 1:** URH-FRM mix design.

Water (kg/m^3^)	Binder (kg/m^3^)					Silica Sand (kg/m^3^)	Set Retarder (kg/m^3^)	VMA (kg/m^3^)	HRWRA (B%)	PE (V%)
	URHC	GGBFS	LSP	CKD	SF					
314	709.4	354.7	177.4	106.4	141.9	496.6	7.09	3.89	1–2%	1.5

Note: URHC, ultra-rapid-hardening cement; GGBFS, ground granulated blast-furnace slag; LSP, limestone powder; CKD, cement kiln dust; SF, silica fume; VMA, viscosity modifying agent; and HRWRA, high-range water-reducing admixture.

**Table 2:** Properties of PE fiber.

Diameter (µm)	Length (mm)	Density (g/m^3^)	Tensile Strength (MPa)	Elastic Modulus (GPa)
30	18	0.97	3000	100

**Table 3:** Experimental variables.

Test	Specimens	Size (mm) (W × D × H)	Reinforcing Material and Thickness
Compressive strength	CR	190 × 90 × 191	Reference (Unreinforced)
	CM10		General mortar 10 mm
	CH10		URH-FRM 10 mm
	CH20		URH-FRM 20 mm
	CH30		URH-FRM 30 mm
Initial shear strength	SR	190 × 90 × 191	Reference (Unreinforced)
	SM10		General mortar 10 mm
	SH10		URH-FRM 10 mm
	SH20		URH-FRM 20 mm
	SH30		URH-FRM 30 mm

**Table 4:** Results of compressive strength test of masonry prism.

Specimen	#1	#2	#3	Average	SD	CV
CR	33.4	33.0	34.3	33.6	0.7	0.02
CM10	32.5	37.8	29.8	33.4	4.1	0.12
CH10	35.4	41.9	28.4	35.2	6.8	0.19
CH20	30.1	28.6	27.7	28.8	1.2	0.04
CH30	38.9	35.6	59.8	44.8	13.1	0.29

Note: SD, standard deviation; CV, coefficients of variation (unit: Mpa).

## Data Availability

Not applicable.
